# *Campylobacter fetus* subspecies *venerealis* meningitis associated with a companion dog in a young adult: a case report

**DOI:** 10.1186/s12879-021-07007-5

**Published:** 2021-12-27

**Authors:** Yeol Jung Seong, Seung Hun Lee, Eun Jin Kim, Young Hwa Choi, Tae-Joon Kim, Wee Gyo Lee, Jung Yeon Heo

**Affiliations:** 1 Department of Infectious Diseases, Ajou University School of Medicine, 164 Worldcup-ro, Youngtong-gu, Suwon, Gyeonggi-do 16499 Republic of Korea; 2 Division of Infectious Disease Diagnosis Control, Honam Regional Center for Disease Control and Prevention, Korea Disease Control and Prevention Agency, Gwangju, Republic of Korea; 3 Department of Neurology, Ajou University School of Medicine, Suwon, Republic of Korea; 4 Department of Laboratory Medicine, Ajou University School of Medicine, 164 Worldcup-ro, Youngtong-gu, Suwon, Gyeonggi-do 16499 Republic of Korea

**Keywords:** Bacterial meningitis, *Campylobacter fetus* subsp. *venerealis*, Companion animal, Zoonotic infectious diseases

## Abstract

**Background:**

Campylobacter spp., common commensals in the gastrointestinal tract of animals, especially poultry, can cause acute gastrointestinal illness in humans through animal-to-human transmission. Although *Campylobacter fetus,* especially subspecies *fetus,* rarely leads to systemic infections such as bacteremia in immunocompromised patients, it is unclear whether *Campylobacter fetus* subspecies *venerealis* (Cfv) causes infectious diseases in humans.

**Case presentation:**

A 28-year-old man with a history of chronic alcoholism visited the emergency department with weakness of the left extremities. The patient was clinically diagnosed with community-acquired bacterial meningitis. The organism from the blood culture was subsequently identified as *Campylobacter fetus.* On phylogenetic analysis, the 16S rRNA sequence showed 99.93% similarity with other Cfv 16S rRNA sequences. The patient had no exposure to identifiable sources except for close contact with a companion dog, which could have been a possible source of transmission.

**Conclusions:**

This case suggests that Cfv could lead to human systemic infections such as meningitis and that companion animals, in addition to well-known animal hosts, could be sources of transmission.

**Supplementary Information:**

The online version contains supplementary material available at 10.1186/s12879-021-07007-5.

## Background

Campylobacter spp., a zoonotic pathogen found in a wide range of animals whose primary reservoirs are the intestinal tracts, usually cause diarrheal illness in humans [1]. Although the vast majority of cases of Campylobacter infection in humans are caused by *Campylobacter jejuni* or *Campylobacter coli*, *Campylobacter fetus* occasionally causes extraintestinal infections, such as bloodstream infection, rather than enteric disease [2]. Invasive *C. fetus* infection has a broad spectrum of clinical presentation from bloodstream infection without apparent localized infection to various types of localized infections, including infection of central nervous system (CNS), osteomyelitis, lung abscess, arthritis, and perinatal infection. Human *C. fetus* infection is uncommon and usually occurs in patients with immunosuppressed conditions or underlying diseases such as cardiovascular disease with valve abnormalities, liver disease, and diabetes mellitus. Two major subspecies of *C. fetus* have been described: *C. fetus* subsp. *fetus* (Cff) and *C. fetus* subsp. *venerealis* (Cfv) [3]. Human infections with a new subspecies of *C. fetus* were proposed to be caused by *C. fetus* subsp. *testudinum*, which has a reptilian origin [4]. Almost nearly all cases of *C. fetus* infection in humans are known to be caused by Cff, and little is known about human infections caused by Cfv. We recently encountered a case of bacterial meningitis caused by Cfv in a young adult. The patient strongly denied having been in close contact with domestic animals or ingesting raw animal products. The route of transmission was suspected to be the frequent close contact with a companion dog, such as while kissing. We also performed a systematic review to enhance our understanding of human *C. fetus* infections of the central nervous system (CNS).

## Case presentation

A 28-year-old man with a history of chronic alcoholism visited the emergency department owing to weakness in the left extremities. He was under treatment with an antiepileptic drug for the past 6 months since a traumatic subdural hemorrhage occurred during a fall down the stairs. At that time, he underwent facial nerve decompression for left-sided facial palsy due to ear bleeding and a temporal bone fracture. He complained of a 5-day history of myalgia and upper respiratory infection symptoms, such as coughing and sore throat. Initially, his vital signs were stable except for a body temperature of 38.6 °C. He was responsive to the medical staff’s questions, but his answers lacked fluency. Findings of a physical examination performed on arrival to the emergency room were unremarkable, and mild neck stiffness was observed during a neurological examination.

Baseline laboratory data of complete blood count showed leukocytosis (white blood cell [WBC] count, 15,090/µL; neutrophils, 87.5%; lymphocytes, 4.9%) with mild C-reactive protein elevation (2.08 mg/dL). Since the patient’s neck stiffness worsened and stupor was noted, a cerebrospinal fluid (CSF) analysis was performed. The CSF analysis showed pleocytosis (WBC count of 390/µL; polymorphonuclear leukocyte count, 60%), high protein level (161.3 mg/dL; reference range, 15–40 mg/dL), and low glucose level (30 mg/dL; reference range, 40–70 mg/dL) with a negative Gram stain. No focal lesions were observed on brain magnetic resonance imaging (Additional file 1: Fig. S1). Intravenous ceftriaxone (2 g every 12 h) and vancomycin (1 g every 12 h) as empirical antibiotic therapy were administered with intravenous dexamethasone, as the findings were suggestive of bacterial meningitis caused by *Streptococcus pneumoniae* or *Neisseria meningitidis*.

On day 6 of hospitalization, blood culture revealed Gram-negative bacilli growth in the aerobic and anaerobic bottles. On day 11 of hospitalization, the organism was identified as *C. fetus.* However, these organisms were not identified in the CSF culture. The CSF specimen was collected 6 h after prompt empirical antibiotic treatment. Doripenem (500 mg every 8 h) was administered for 10 days until the meningitis symptoms completely resolved without neurological sequelae. The automated susceptibility test (VITEK2 system, bioMérieux, France) showed that the isolate was susceptible to erythromycin and ciprofloxacin. The patient regularly visited the outpatient clinic for 2 years, without recurrence of the meningitis. He stated that he had been raising a companion dog and denied contact with livestock animals such as cattle and sheep or ingestion of raw or undercooked meat.

**Fig. 1:**
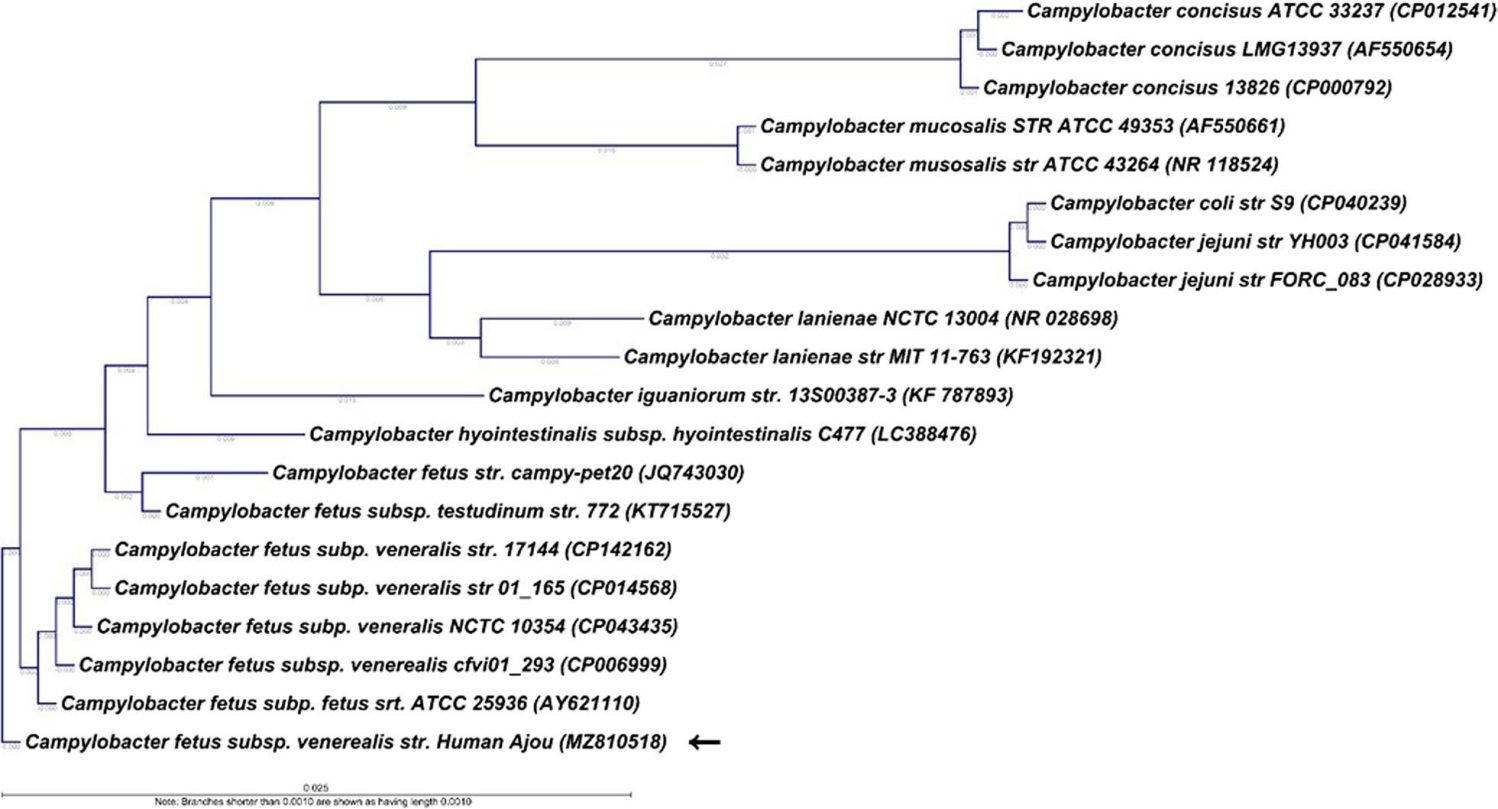
Phylogenetic analysis constructed using Jukes-Cantor/Neighbor Joining methods. The 16S rRNA sequence of *Campylobacter fetus* marked by arrow is compared to published sequences of other *Campylobacter* spp. from GenBank. The 16S rRNA sequence in this case shows high similarity (99.93%) to other *Campylobacter fetus* subsp. *venerealis* 16S rRNA sequences

**Fig. 2:**
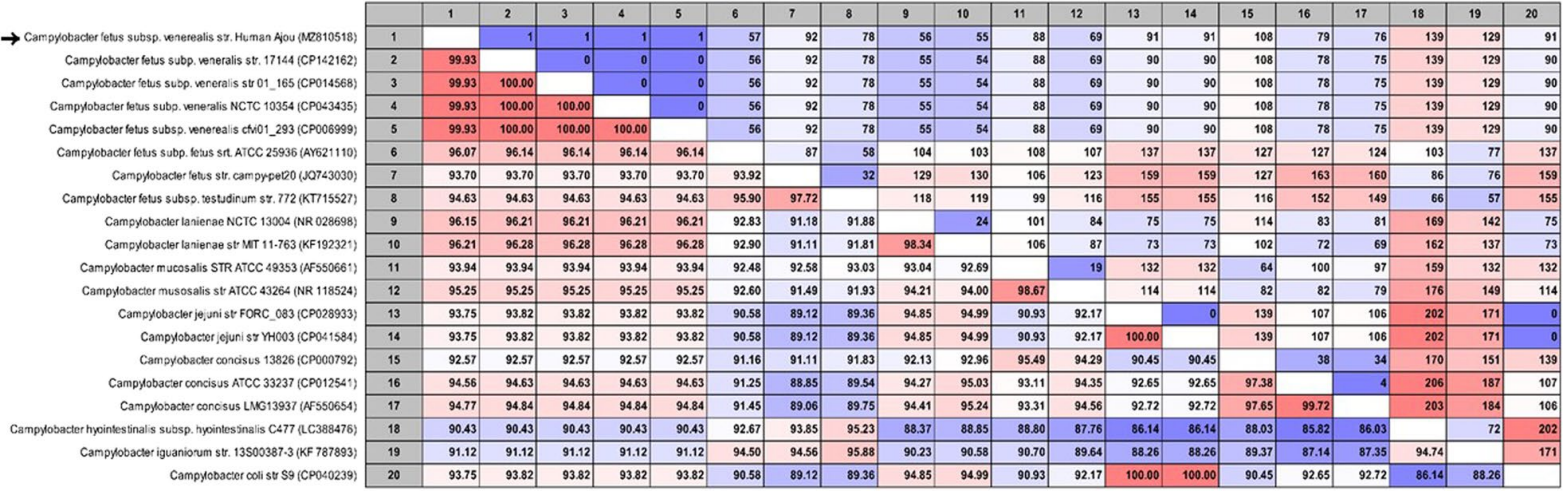
Previously reported 16S rRNA nucleotides of other *Campylobacter* species. The 16S rRNA sequences of *Campylobacter fetus* subsp. *venerealis* marked by arrows are distinct from those of *C. fetus* subsp. *fetus*

To confirm the species and identify the subspecies of the isolated *C. fetus* sample, a sequence analysis of the 16 s rRNA gene was conducted using the polymerase chain reaction primers 27F 5′-AGA GTT TGA TCM TGG CTC-3′ and 1492R 5′-TAC GGY TAC CTT GTT ACG ACT-3′. The sequencing primers 785F 5′ (GGA TTA GAT ACC CTG GTA) 3' and 907R 5′ (CCG TCA ATT CMT TTR AGT TT) 3′ were used. The *C. fetus* 16S rRNA sequence was compared to the published sequences from GenBank. Phylogenetic analysis (Jukes-Cantor/Neighbor Joining) revealed that the 16S rRNA sequence in this case showed high similarity (99.93%) with other Cfv 16S rRNA sequences (Fig. 1). These sequences were also distinct from those of Cff and other Campylobacter spp. compared with previously reported 16S rRNA nucleotides of other Campylobacter species (*C, coli*, *C. jejuni*, Cff) (Fig. 2).

## Discussion and conclusions

Here we described a case of *C. fetus* meningitis in a young adult with a history of heavy alcoholism who was being treated with an antiepileptic drug for a traumatic cerebral hemorrhage. A culture isolate of *C. fetus* was identified as Cfv by sequence analysis of the 16s rRNA gene. *C. fetus* is divided into two major subspecies: Cff and Cfv [3]. These subspecies are genetically closely related but have different habitats and clinical importance. The primary reservoir of Cff is the intestinal tract of cattle and sheep [5]. Cff is a clinically significant pathogenic organism in animals and an opportunistic pathogen in humans. It has been identified in human cases of bloodstream infection, vascular infection, and cellulitis in addition to meningitis [6, 7]. In contrast, Cfv is a commensal organism of the bovine genital tract that causes an infectious disease known as bovine genital campylobacteriosis, which leads to infertility and enzootic abortion in cattle, resulting in considerable economic losses [8]. Cfv has been isolated from human specimens in only a few cases [9], and its clinical significance remains uncertain. Thus, this is a rare case of systemic human infection caused by Cfv*.*

To perform a systematic review for *Campylobacter fetus* meningitis, the MEDLINE database was searched using keywords *Campylobacter fetus* AND meningitis, *Vibrio fetus* AND meningitis, and *Spirillum serpens* AND meningitis. Literature written in English, French, German, Spanish, Japanese, and Korean were included. Case descriptions of animals or children were excluded. We identified 38 cases of CNS infections in 34 related articles published since 1960 (Table 1). Among these patients, 30 were men (78.9%). The median age was 49.5 years (interquartile range, 39.8–57.0 years). Immunocompromised conditions were observed in 24 cases (63.2%), and the prevalent underlying conditions were alcoholism (14 cases [36.8%]) and diabetes (6 cases [15.8%]). Although *C. fetus* is a zoonotic pathogen, the potential source of infection, such as animal or animal product contact, was identified in only 15 cases (39.5%). Among the identified cases, the likely source of infection and the major risk factors for exposure to *C. fetus* were the ingestion of raw or undercooked meat (6 cases [40.0%]) and frequent contact with animals (5 cases [33.3%]).

**Table 1:** A case summary of *Campylobacter fetus* meningitis based on a systematic literature review

Year^Ref^*	Age/Sex	Underlying condition	Source of infection	Specimens	(Sub)species	Clinical manifestations	Treatment	Outcome
2021	33/F	ALL	Undercooked beef ingestion	Blood and CSF	Cf	Meningitis	MER	Recovery
2019	56/M	Chronic alcoholism	Unknown	CSF	Cf	Meningoencephalitis	AMP	Recovery
2019	35/F	No	Unknown	Blood	Cf	Meningitis and spondylodiscitis	MEP, AMP	Recovery
2018	48/F	No	Raw beef and cattle liver ingestion	CSF	Cff/Cfv	Meningitis	CRO	Recovery
2017	64/M	Alcoholic liver cirrhosis and diabetes	Unknown	Blood	Cff	Meningitis	DOR	Recovery
2016	23/F	No	Domestic animals, worked on a farm	CSF	Cf	Meningitis	CRO, MER	Cured after relapse, cognitive defect
2016	52/M	No	Farmer	Blood and CSF	Cff	Meningitis	CRO, MER	Cured after relapse
2013	75/M	Diabetes	Raw sheep liver ingestion	Blood and CSF	Cff	Meningitis and endocarditis	IPM, GEN	Recovery
2013	28/M	Seizure disorder	Khat chewing	Blood	Cff	Meningitis	CRO	Recovery
2009	40/M	Crohn’s disease	Unknown	Blood, CSF, and stool	Cf	Meningitis	PIP	Recovery
2008	51/M	No	Unknown	Blood and CSF	Cff	Subdural empyema	NA	Recovery
2006	43/M	No	Unknown	CSF	Cf	Meningitis	MER	Recovery
2004	71/M	Diabetes	Unknown	CSF	Cff	Meningitis	IPM	Recovery
2002	49/M	Chronic alcoholism	Unknown	Blood and CSF	Cff	Meningoencephalitis and spondylodiscitis	NA	Recovery
1998	47/M	Chronic alcoholism	Dog and cat	Blood	Cff	Meningitis	CTX, OFX, GEN	Recovery
1997	70/M	Chronic alcoholism	Unknown	CSF	Cf	Infected subdural hematoma	IPM	Recovery
1996	84/M	Alcoholic liver cirrhosis	Unknown	Blood and CSF	Cf	Meningitis	CRO, CIP	Died
1993	40/M	No	Raw beef	Blood and CSF	Cff	Meningitis	IPM	Recovery
1990	55/M	Chronic alcoholism and diabetes	Unknown	CSF	Cf	Meningitis	AMP	Recovery
1989	39/F	Chronic alcoholism, epilepsy	Unknown	Blood and CSF	Cff	Meningitis	AMS	Recovery
1989	36/M	Chronic alcoholism	Unknown	Blood	Cff	Meningitis	AMP	Recovery
1987	47/M	Kidney transplantation recipient	Raw cattle liver ingestion	Blood and CSF	Cfi	Meningitis	ERY, CHL	Recovery
1986	30/M	No	Raw cattle liver ingestion	CSF	Cff	Meningitis	AMP	Recovery
1986	42/M	No	Unknown	CSF	Cff	Meningitis	MIN	Recovery
1985	68/M	Rectal cancer with hepatic metastasis	Unknown	Blood and CSF	Cff	Meningitis	CFZ, TOB, ERY, AMP, GEN	Died
1985	65/M	Alcoholic liver cirrhosis	Unknown	Blood	Cff	Meningitis	ERY	Cured after relapse
1985	38/M	Chronic alcoholism	Cat	CSF	Cff	Meningitis	AMP, GEN	Recovery
1984	53/M	No	Unknown	Blood and CSF	Cff	Meningitis	CHL	Recovery
1980	34/M	No	Unknown	CSF	Cfj	Meningitis	CHL	Recovery
1978	50/M	No	Contact with uncooked meat	Blood	Vf	Meningitis	AMP, CHL	Recovery
1976	40/M	No	Frequent contact with domestic animal	CSF	Cfi	Meningitis	ERY, STM	Recovery
1971	53/M	Chronic alcoholism	Unknown	Blood and CSF	Vf	Meningoencephalitis	AMP, KAN	Comatose mentality
1969	50/M	Diabetes	Unknown	CSF	Vf	Meningitis	PEN, AMP, CHL	Recovery
1969	69/F	Diabetes, ITP	Unknown	Blood and CSF	Vf	Meningitis	PEN, CHL, SFZ	Died
1966	48/F	No	Farmer, cared for sick calves	Blood and pericardial fluid	Vf	Pericarditis and meningitis	PEN, CHL	Hemiparesis
1964	55/M	CLL	Rats at workplace	Blood and CSF	Vf	Meningitis	PEN, TET	Recovery
1962	47/M	Chronic alcoholism	Unknown	Blood and CSF	Vf	Meningitis	PEN, TET	Recovery
1960	50/F	Chronic alcoholism	Lived in rat-infested neighborhood	Blood and CSF	Ss	Meningitis	PEN, CHL	Recovery

^*^References for *Campylobacter fetus* meningitis are presented as Additional file 1

ALL, acute lymphoblastic leukemia; AMP, ampicillin; AMS, ampicillin/sulbactam; Cf, *Campylobacter fetus*; Cff, *Campylobacter fetus* subspecies *fetus*; Cfi, *Campylobacter fetus* subspecies *intestinalis*; Cfj, *Campylobacter fetus* subspecies *jejuni*; Cfv, *Campylobacter fetus* subspecies *venerealis*; CFZ, cefazolin; CHL, chloramphenicol; CIP, ciprofloxacin; CLL, chronic lymphocytic leukemia; CRO, ceftriaxone; CSF, cerebrospinal fluid; CTX, cefotaxime; DOR, doripenem; ERY, erythromycin; GEN, gentamicin; IPM, imipenem; ITET, tetracycline; KAN, kanamycin; MEP, meropenem; MIN, minocycline; NA, not available; OFX, ofloxacin; PEN, penicillin; PIP, piperacillin; Ref, references; SFZ, sulfadiazine; Ss, *Spirillum serpens*; STM, streptomycin; TOB, tobramycin; TP, immune thrombocytopenic purpura; Vf, *Vibrio fetus*

This case shared common features of chronic alcoholism and frequent animal contact with the findings of the systematic review. In addition, the previous subdural hemorrhage in this case may have been a predisposing factor for meningitis. Potential disruption of the blood–brain barrier could be a pathway for microbes to invade the central nervous system. However, other potential sources of infection were not identified, except for contact with the companion dog. We were unable to demonstrate that the dog was the source of transmission; however, given that *C. jejuni* transmission from a companion dog was genetically proven previously [10], the same is certainly plausible in this case. This case suggests that companion dogs could be a reservoir for zoonotic pathogens and their owners should be educated on zoonotic disease risk and prevention.

Systemic *C. fetus* infections, such as sepsis or meningitis, should be treated with parenteral antibiotics. Through a systematic review, we identified that most of the recently reported cases of *C. fetus* meningitis were treated with carbapenem antibiotics, similar to the present case. However, *C. fetus* is generally susceptible to ampicillin, cefotaxime, ciprofloxacin, aminoglycoside, and imipenem but not erythromycin [11]. Despite these patterns of antimicrobial susceptibility, *C. fetus* infection in the central nervous system should be treated with prolonged antibiotic treatment for at least 2–3 weeks [1]. The surface-layer proteins of *C. fetus*, critical factors in its virulence that form a capsule-like structure, can undergo antigenic variation that enables evasion of the host’s immune system [12]. Therefore, an invasive *C. fetus* infection could relapse or persist even several years after the initial episode.

To the best of our knowledge, human Cfv infection has rarely been reported: We found only one case of an adult with meningitis and five cases of patients with bacteremia [9, 13]. These human cases with Cfv infection were mainly identified by 16S rRNA sequencing analysis. However, it could be difficult to differentiate between subspecies *fetus* and *venerealis* because of the modest subspecies-specific variation at the genome level [14]. A case of meningitis revealed that Cff and Cfv were verified through matrix-assisted laser desorption/ionization-time of flight mass spectrometry (MALI-TOF-MD) and 16S rRNA sequencing. Although five cases of bacteremia were identified as Cfv on the 16S rRNA sequencing analysis, three cases were identified as Cff and the other two cases were identified as Cfv by the multiplex PCR method using the *cad*F, *hip*O, and *asp* genes. Other bacteremia or meningitis cases attributed to *C. fetus* subspecies were mostly caused by Cff*.* The present case demonstrated that Cfv isolated from the blood has sequences distinct from Cff based on the 16S rRNA sequence and phylogenetic analysis.

Our case suggests that Cfv could cause human systemic infections, such as meningitis, and may be associated with companion animals in addition to well-known animal hosts.

## Supplementary Information

**Additional file 1.** Supplementary material.

## Data Availability

The 16S rRNA sequences of *Campylobacter fetus* subsp. *venerealis* are available in the GenBank database (Accession Number: MZ810518).

## Abbreviations

ALL: Acute lymphoblastic leukemia
AMP: Ampicillin
AMS: Ampicillin/sulbactam
*C. coli*: *Campylobacter coli*
*C. fetus*: *Campylobacter fetus*
*C. jejuni*: *Campylobacter jejuni*
Cf: *Campylobacter fetus*
Cff: *Campylobacter fetus* Subspecies *fetus*
Cfi: *Campylobacter fetus* Subspecies *intestinalis*
Cfj: *Campylobacter fetus* Subspecies *jejuni*
Cfv: *Campylobacter fetus* Subspecies *venerealis*
CFZ: Cefazolin
CHL: Chloramphenicol
CIP: Ciprofloxacin
CLL: Chronic lymphocytic leukemia
CNS: Central nerve system
CRO: Ceftriaxone
CSF: Cerebrospinal fluid
CTX: Cefotaxime
DOR: Doripenem
ERY: Erythromycin
GEN: Gentamicin
IPM: Imipenem
ITET: Tetracycline
KAN: Kanamycin
MEP: Meropenem
MIN: Minocycline
NA: Not available
OFX: Ofloxacin
PEN: Penicillin
PIP: Piperacillin
Ref: References
rRNA: Ribosomal ribonucleic acid
SFZ: Sulfadiazine
WBC: White blood cell
