# Supplementary material for: Campylobacter fetus subspecies venerealis meningitis associated with a companion dog in a young adult: a case report
Source: BMC Infect Dis. 2021 Dec 27;21:1280. doi: 10.1186/s12879-021-07007-5 (PMC8711199; doi:10.1186/s12879-021-07007-5)
Supplement: Supplementary file 1 — Additional file 1. Supplementary material. [file 12879_2021_7007_MOESM1_ESM.docx]

**Supplementary Figure 1.** Magnetic resonance imaging on hospital admission. (A) Diffusion-weighted imaging and (B) T1-weighted imaging with gadolinium show encephalomalatic change and old hemosidern deposit with thin enhancement on the right side of frontal and temporal lobe due to previous traumatic sequelae. There is no clear evidence suggestive of meningoencephalitis.


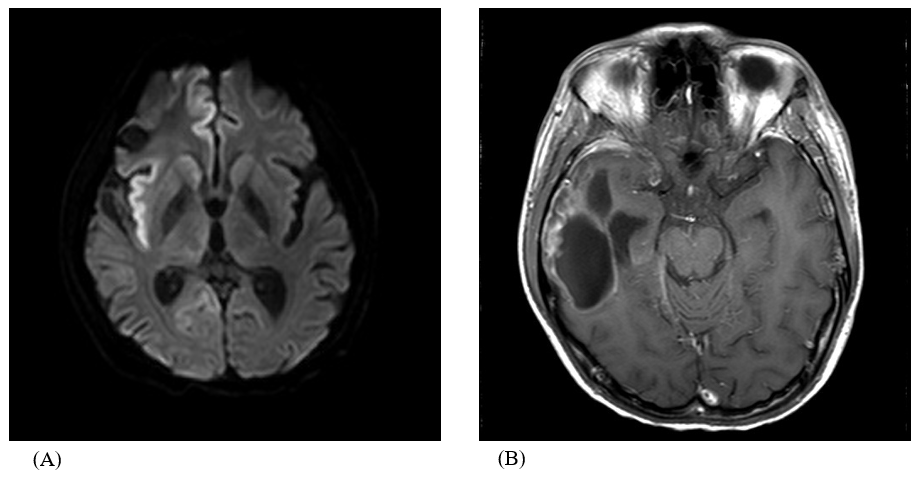


**Supplementary Table 1.** References for cases of *Campylobacter fetus* meningitis

| Year^Ref✻^ | Age/Sex | Underlying condition | Source of infection | Specimens | (Sub)species | Clinical manifestations | Treatment | Outcome |
| --- | --- | --- | --- | --- | --- | --- | --- | --- |
| 2021 [1] | 33/F | ALL | Undercooked beef ingestion | Blood and CSF | Cf | Meningitis | MER | Recovery |
| 2019 [2] | 56/M | Chronic alcoholism | Unknown | CSF | Cf | Meningoencephalitis | AMP | Recovery |
| 2019 [3] | 35/F | No | Unknown | Blood | Cf | Meningitis and spondylodiscitis | MEP, AMP | Recovery |
| 2018 [4] | 48/F | No | Raw beef and cattle liver ingestion | CSF | Cff/Cfv | Meningitis | CRO | Recovery |
| 2017 [5] | 64/M | Alcoholic liver cirrhosis and diabetes | Unknown | Blood | Cff | Meningitis | DOR | Recovery |
| 2016 [6] | 23/F | No | Domestic animals, worked on a farm | CSF | Cf | Meningitis | CRO, MER | Cured after relapse, cognitive defect |
| 2016 [6] | 52/M | No | Farmer | Blood and CSF | Cff | Meningitis | CRO, MER | Cured after relapse |
| 2013 [7] | 75/M | Diabetes | Raw sheep liver ingestion | Blood and CSF | Cff | Meningitis and endocarditis | IPM, GEN | Recovery |
| 2013 [8] | 28/M | Seizure disorder | Khat chewing | Blood | Cff | Meningitis | CRO | Recovery |
| 2009 [9] | 40/M | Crohn’s disease | Unknown | Blood, CSF, and stool | Cf | Meningitis | PIP | Recovery |
| 2008 [10] | 51/M | No | Unknown | Blood and CSF | Cff | Subdural empyema | NA | Recovery |
| 2006 [11] | 43/M | No | Unknown | CSF | Cf | Meningitis | MER | Recovery |
| 2004 [12] | 71/M | Diabetes | Unknown | CSF | Cff | Meningitis | IPM | Recovery |
| 2002 [13] | 49/M | Chronic alcoholism | Unknown | Blood and CSF | Cff | Meningoencephalitis and spondylodiscitis | NA | Recovery |
| 1998 [14] | 47/M | Chronic alcoholism | Dog and cat | Blood | Cff | Meningitis | CTX, OFX, GEN | Recovery |
| 1997 [15] | 70/M | Chronic alcoholism | Unknown | CSF | Cf | Infected subdural hematoma | IPM | Recovery |
| 1996 [16] | 84/M | Alcoholic liver cirrhosis | Unknown | Blood and CSF | Cf | Meningitis | CRO, CIP | Died |
| 1993 [17] | 40/M | No | Raw beef | Blood and CSF | Cff | Meningitis | IPM | Recovery |
| 1990 [18] | 55/M | Chronic alcoholism and diabetes | Unknown | CSF | Cf | Meningitis | AMP | Recovery |
| 1989 [19] | 39/F | Chronic alcoholism, epilepsy | Unknown | Blood and CSF | Cff | Meningitis | AMS | Recovery |
| 1989 [19] | 36/M | Chronic alcoholism | Unknown | Blood | Cff | Meningitis | AMP | Recovery |
| 1987 [20] | 47/M | Kidney transplantation recipient | Raw cattle liver ingestion | Blood and CSF | Cfi | Meningitis | ERY, CHL | Recovery |
| 1986 [21] | 30/M | No | Raw cattle liver ingestion | CSF | Cff | Meningitis | AMP | Recovery |
| 1986 [21] | 42/M | No | Unknown | CSF | Cff | Meningitis | MIN | Recovery |
| 1985 [22] | 68/M | Rectal cancer with hepatic metastasis | Unknown | Blood and CSF | Cff | Meningitis | CFZ, TOB, ERY, AMP, GEN | Died |
| 1985 [22] | 65/M | Alcoholic liver cirrhosis | Unknown | Blood | Cff | Meningitis | ERY | Cured after relapse |
| 1985 [23] | 38/M | Chronic alcoholism | Cat | CSF | Cff | Meningitis | AMP, GEN | Recovery |
| 1984 [24] | 53/M | No | Unknown | Blood and CSF | Cff | Meningitis | CHL | Recovery |
| 1980 [25] | 34/M | No | Unknown | CSF | Cfj | Meningitis | CHL | Recovery |
| 1978 [26] | 50/M | No | Contact with uncooked meat | Blood | Vf | Meningitis | AMP, CHL | Recovery |
| 1976 [27] | 40/M | No | Frequent contact with domestic animal | CSF | Cfi | Meningitis | ERY, STM | Recovery |
| 1971 [28] | 53/M | Chronic alcoholism | Unknown | Blood and CSF | Vf | Meningoencephalitis | AMP, KAN | Comatose mentality |
| 1969 [29] | 50/M | Diabetes | Unknown | CSF | Vf | Meningitis | PEN, AMP, CHL | Recovery |
| 1969 [30] | 69/F | Diabetes, ITP | Unknown | Blood and CSF | Vf | Meningitis | PEN, CHL, SFZ | Died |
| 1966 [31] | 48/F | No | Farmer, cared for sick calves | Blood and pericardial fluid | Vf | Pericarditis and meningitis | PEN, CHL | Hemiparesis |
| 1964 [32] | 55/M | CLL | Rats at workplace | Blood and CSF | Vf | Meningitis | PEN, TET | Recovery |
| 1962 [33] | 47/M | Chronic alcoholism | Unknown | Blood and CSF | Vf | Meningitis | PEN, TET | Recovery |
| 1960 [34] | 50/F | Chronic alcoholism | Lived in rat-infested neighborhood | Blood and CSF | Ss | Meningitis | PEN, CHL | Recovery |

ALL, acute lymphoblastic leukemia; AMP, ampicillin; AMS, ampicillin/sulbactam; Cf, *Campylobacter fetus*; Cff, *Campylobacter fetus* subspecies *fetus*; Cfi, *Campylobacter fetus* subspecies *intestinalis*; Cfj, *Campylobacter fetus* subspecies *jejuni*; Cfv, *Campylobacter fetus* subspecies *venerealis*; CFZ, cefazolin; CHL, chloramphenicol; CIP, ciprofloxacin; CLL, chronic lymphocytic leukemia; CRO, ceftriaxone; CSF, cerebrospinal fluid; CTX, cefotaxime; DOR, doripenem; ERY, erythromycin; GEN, gentamicin; IPM, imipenem; ITET, tetracycline; KAN, kanamycin; MEP, meropenem; MIN, minocycline; NA, not available; OFX, ofloxacin; PEN, penicillin; PIP, piperacillin; Ref, references; SFZ, sulfadiazine; Ss, *Spirillum serpens*; STM, streptomycin; TOB, tobramycin; TP, immune thrombocytopenic purpura; Vf, *Vibrio fetus*

[1] Nakatani R, Shimizu K, Matsuo T, Koyamada R, Mori N, Yamashita T, et al. *Campylobacter fetus* bacteremia and meningitis in an acute lymphoblastic leukemia patient undergoing maintenance therapy: a case report. BMC Infect Dis 2021;21:680.

[2] Tanabe S, Kutsuna S, Tsuboi M, Takeshita N, Hayakawa K, Ohmagari N. Meningoencephalitis Caused by a *Campylobacter fetus* in a Patient with Chronic Alcoholism. Intern Med 2019;58:2247-50.

[3] Ikeda K, Manabe Y, Fujiwara S, Omote Y, Narai H, Abe K. *Campylobacter fetus* Meningitis and Pyogenic Spondylodiscitis in a Healthy Young Woman. Case Rep Neurol 2019;11:299-303.

[4] Ishihara A, Hashimoto E, Ishioka H, Kobayashi H, Gomi H. *Campylobacter fetus* meningitis associated with eating habits of raw meat and raw liver in a healthy patient: A case report and literature review. IDCases 2018;11:97-100.

[5] Moon J, Kim N, Lee HS, Shin HR, Lee ST, Jung KH, et al. *Campylobacter fetus* meningitis confirmed by a 16S rRNA gene analysis using the MinION nanopore sequencer, South Korea, 2016. Emerg Microbes Infect 2017;6:e94.

[6] van Samkar A, Brouwer MC, van der Ende A, van de Beek D. *Campylobacter fetus* Meningitis in Adults: Report of 2 Cases and Review of the Literature. Medicine (Baltimore) 2016;95:e2858.

[7] Suy F, Le Dû D, Roux AL, Hanachi M, Dinh A, Crémieux AC. Meningitis and endocarditis caused by *Campylobacter fetus* after raw-liver ingestion. J Clin Microbiol 2013;51:3147-50.

[8] Martínez-Balzano C, Kohlitz PJ, Chaudhary P, Hegazy H. *Campylobacter fetus* bacteremia in a young healthy adult transmitted by khat chewing. J Infect 2013;66:184-6.

[9] Umehara Y, Kudo M, Kawasaki M. *Campylobacter fetus* meningitis in a patient with Crohn's disease. Inflamm Bowel Dis 2009;15:645-6.

[10] Kanayama S, Ohnishi K, Yamaura T, Katayama M, Makino J, Takemura N, et al. Case of bilateral subdural empyema complicating *Campylobacter fetus* subspecies *fetus* meningitis. Brain Nerve 2008;60:659-62.

[11] Shioyama M, Mitui Y, Ueda H, Takada K, Kureshiro J, Kitaguchi M, et al. Bacterial meningitis with *Campylobacter fetus* manifesting chronic clinical course. Rinsho Shinkeigaku 2006;46:699-701.

[12] Herve J, Aissa N, Legrand P, Sorkine M, Calmette MJ, Santin A, et al. *Campylobacter fetus* meningitis in a diabetic adult cured by imipenem. Eur J Clin Microbiol Infect Dis 2004;23:722-4.

[13] Ozeki T, Nokura K, Koga H, Yamamoto H. A case of meningoencephalitis and spondylodiscitis caused by *Campylobacter fetus* subsp. *fetus* infection. Rinsho Shinkeigaku 2002;42:38-41.

[14] Dronda F, García-Arata I, Navas E, de Rafael L. Meningitis in adults due to *Campylobacter fetus* subspecies *fetus*. Clin Infect Dis 1998;27:906-7.

[15] Aoki N, Sakai T, Oikawa A, Takizawa T, Shishido T. Infected subdural effusion associated with resolving subdural hematoma--case report. Neurol Med Chir (Tokyo) 1997;37:637-9.

[16] Wilhelm JM, Saraceni O, Penner MF, Trévoux A, Kieffer P. *Campylobacter fetus* meningitis in adults. Presse Med 1996;25:1331-2.

[17] Inoue Y, Ohtsubo T, Mori N, Ishino T, Takase T, Kaku M, et al. A case of *Campylobacter fetus* subspecies *fetus* meningitis. Kansenshogaku Zasshi 1993;67:66-70.

[18] Kato H, Wakasugi H, Mukuta T, Furukawa M, Yokota M, Yamada Y, et al. *Campylobacter fetus* subspecies *fetus* meningitis with chronic alcoholism and diabetes mellitus. Jpn J Med 1990;29:542-4.

[19] Clavelou P, Beytout J, Gourdiat A, Garandeau A, Deffond D, Tournilhac M. Neurologic involvement in campylobacter infections. 5 cases. Rev Neurol (Paris) 1989;145:208-14.

[20] Rao KV, Ralston RA. Meningitis due to *Campylobacter fetus intestinalis* in a kidney transplant recipient. A case report. Am J Nephrol 1987;7:402-3.

[21] Iida Y, Sunaga K, Maehara K, Okamoto Y, Yasunaga K, Nishiura K, et al. Two cases of *Campylobacter fetus meningitis*. Kansenshogaku Zasshi 1986;60:271-6.

[22] Francioli P, Herzstein J, Grob JP, Vallotton JJ, Mombelli G, Glauser MP. *Campylobacter fetus* subspecies *fetus* bacteremia. Arch Intern Med 1985;145:289-92.

[23] Malbrunot C, Zelinsky A, Genevray B, Debenes B, Dechy H, Dorra M. Meningitis caused by *Campylobacter fetus fetus*. A case report. Presse Med 1985;14:1608.

[24] Hanai N, Odawara H, Masuda G, Yano Y, Saku K. *Campylobacter fetus* meningitis in an adult male. Kansenshogaku Zasshi 1984;58:441-6.

[25] Norrby R, McCloskey RV, Zackrisson G, Falsen E. Meningitis caused by *Campylobacter fetus* ssp *jejuni*. Br Med J 1980;280:1164.

[26] Zelinger KS, Vargas RD. Central nervous system infection by *Vibrio fetus*. Neurology 1978;28:968-71.

[27] Gubina M, Zajc-Satler J, Mehle J, Drinovec B, Pikelj F, Radsel-Medvescek A, et al. Septicaemia and meningitis with *Campylobacter fetus* subspecies *intestinalis*. Infection 1976;4:115-8.

[28] Gunderson CH, Sack GE. Neurology of *Vibrio fetus* infection. Neurology 1971;21:307-9.

[29] Stille W, Helm EB. Sepsis and meningitis caused by *Vibrio fetus*. Dtsch Med Wochenschr 1969;94:2484-8.

[30] Reyman TA, Silberberg B. *Vibrio fetus* septicemia. Am J Clin Pathol 1969;51:578-83.

[31] Killam HA, Crowder JG, White AC, Edmonds JH, Jr. Pericarditis due to *Vibrio fetus*. Am J Cardiol 1966;17:723-8.

[32] Collins HS, Blevins A, Benter E. Protracted bacteremia and meningitis due to *Vibrio fetus*. Arch Intern Med 1964;113:361-4.

[33] Robin LA, Duprey G, Jouannot JF, Paris P, Magard H, Mignard J, et al. Apropos of 3 cases of human vibriosis (*Vibrio fetus*), including 1 case of meningitis. Presse Med 1962;70:321-3.

[34] Edwards CE, Kraus R. *Spirillum serpens* meningitis. Report of a case. N Engl J Med 1960;262:458-60.
